# Advances in Transdermal Drug Delivery Systems for Antithrombotic Therapy: A Systematic Review

**DOI:** 10.1021/acsptsci.5c00586

**Published:** 2025-11-04

**Authors:** Maria Augusta D. Stersi, Giovanna C. Nader-Mota, Erika Y. Suzuki, Lucio M. Cabral, Plínio C. Sathler, Flávia A. do Carmo

**Affiliations:** † Department of Drugs and Pharmaceutics, Faculty of Pharmacy, 28125 Universidade Federal do Rio de Janeiro, Rio de Janeiro 21941-902, Brazil; ‡ Institute of Drug Technology, Oswaldo Cruz Foundation, Rio de Janeiro 21040-900, Brazil; § Department of Pharmaceutical Sciences, 67825 Universidade Federal Rural do Rio de Janeiro, Seropédica 23897-090, Brazil; △ Department of Clinical and Toxicological Analysis, Faculty of Pharmacy, Universidade Federal do Rio de Janeiro, Rio de Janeiro 21941-902, Brazil

**Keywords:** antithrombotic, thromboembolism, transdermal drug delivery, coagulation, skin penetration

## Abstract

Thrombotic diseases, classified as arterial or venous, remain one of the most important global health concerns. Myocardial infarction, ischemic stroke, and venous thromboembolism (VTE), which include deep vein thrombosis and pulmonary embolism, are prominent causes of illness and death. Antithrombotic agents, classified by their sites of action, are essential for preventing and treating thrombus formation. Transdermal drug delivery systems have emerged as promising alternatives for antithrombotic therapy by improving drug bioavailability, patient adherence, and therapeutic efficacy while reducing side effects. This systematic review, conducted in accordance with the Preferred Reporting Items for Systematic Reviews and Meta-analyses (PRISMA) guidelines, identified 25 relevant articles through structured database searches. An additional search in clinical trial registries revealed no ongoing or completed clinical studies involving transdermal antithrombotic therapy. The literature focuses on transdermal formulations of heparins and acetylsalicylic acid, with fewer reports on direct oral anticoagulants and other agents. The literature search revealed that the most investigated delivery systems were microneedles (13), micro/nanoemulsions (2), ethosomes (1), hydrogels (5), polymeric patches (3), and liposomes (1). The ongoing interest in antithrombotic transdermal formulations highlights both their therapeutic importance and the difficulties still associated with traditional administration methods. While innovative transdermal formulations show promise, further research is necessary to develop scalable, effective, and cost-efficient technologies for clinical applications.

## Graphical abstract

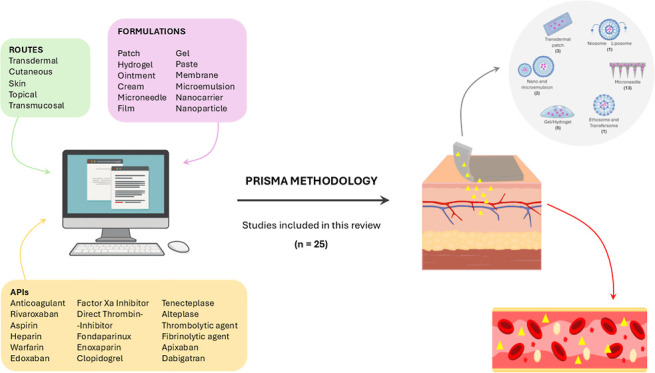

Thrombotic diseases represent one of the most common causes of morbidity and mortality globally, estimated to contribute to one in four deaths, as they serve as the primary pathological process involving myocardial infarction, ischemic stroke, and venous thromboembolism (VTE).^1,2^ This association is grounded in the fact that intravascular thrombus formation can obstruct blood flow, causing tissue hypoxia and necrosis.^3^ In the heart, coronary atherosclerosis predisposes to plaque rupture and platelet activation, leading to thrombus formation, restricted myocardial perfusion, and infarction.^4,5^ In the brain, ischemic stroke results from thrombotic occlusion of cerebral arteries, which abruptly reduces blood supply and causes neuronal death and neurological deficits.^6,7^ Aging appears to be a significant risk factor for thrombosis. As global life expectancy continues to rise, the prevalence of thrombotic diseases has increased accordingly.^8^ Moreover, the Coronavirus Disease 2019 (COVID-19) pandemic has been associated with rising mortality rates over the past years, highlighting even more the significance of thrombosis as a global health concern.^9,10^

The understanding of hemostasis has progressively evolved through successive conceptual models, catalyzed by continuous scientific and technological advances in health research over the past decades.^11^ According to the classic model, hemostasis is divided into primary, secondary, and tertiary phases (Figure 1).

**Figure 1:**
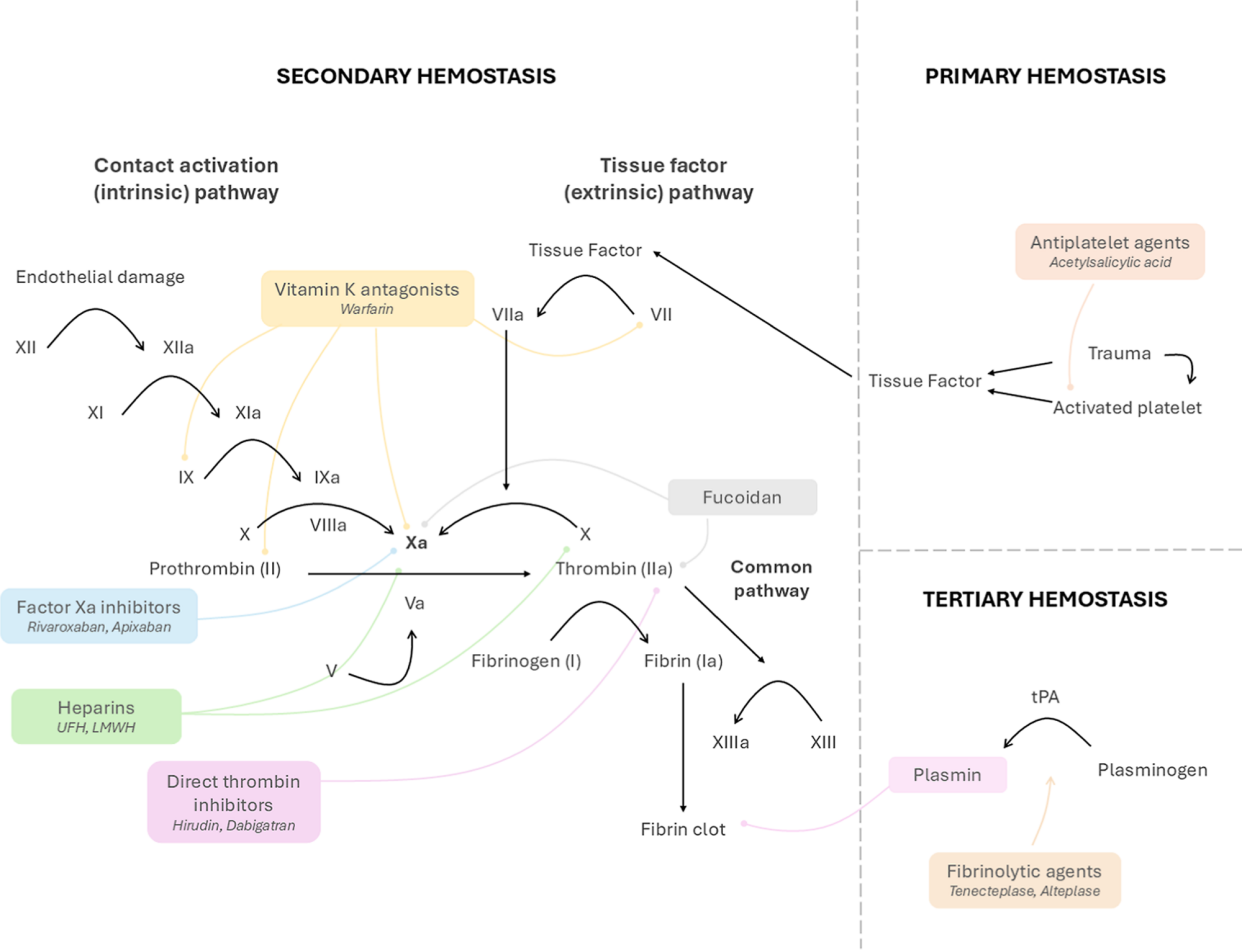
Schematic representation of the primary, secondary, and tertiary hemostasis, highlighting the target sites of antithrombotic drugs.

Primary hemostasis corresponds to the initial response to vascular injury, during which platelets adhere to the damaged endothelium, become activated, and aggregate to form a platelet plug that temporarily seals the lesion. This process is essential for the initial cessation of bleeding. Initially, platelets adhere to the damaged vessel wall through glycoprotein (GP) Ib–IX–V interactions and through collagen receptors such as GP VI and integrin α_2_β_1_.^12^ Activated platelets undergo shape change, release α-granule contents (fibrinogen, factor V, and platelet factor IV), and expose phosphatidylserine (PS) on their outer membrane in a process known as flip-flop, which provides a negatively charged surface for complex assembly.^13^ Concomitantly, dense-granule secretion releases adenosine diphosphate (ADP), serotonin, and calcium ions to recruit and activate surrounding platelets through G protein–coupled receptors (GPCRs), which trigger intracellular signaling cascades that elevate calcium levels, activate protein kinase C, and promote the activation of integrin GP IIb/IIIa, thereby reinforcing platelet aggregation and consolidating the growing platelet.^12^ Platelet activation also stimulates phospholipase A_2_, releasing arachidonic acid from membrane phospholipids, converting this substrate into thromboxane A_2_ (TxA_2_) via the cyclooxygenase (COX) pathway (cyclooxygenase-1—COX-1 and cyclooxygenase-2—COX-2).^14^ TxA_2_ acts through its receptor to further amplify platelet recruitment, vasoconstriction, and stabilization of the developing thrombus.^12^

Subsequently, secondary hemostasis consists of fibrin formation through activation of the coagulation cascade, which, according to the classic coagulation cascade model, includes the intrinsic, extrinsic, and common pathways.^15–17^ Based on the cascade model and insights gained from coagulation enzymology, there were significant advances in the field of anticoagulation, resulting in the identification of therapeutic agents such as heparin and warfarin.^11^ Nevertheless, there were several modifications to the classic coagulation cascade model due to inconsistencies, such as its inability to explain why an intact extrinsic pathway cannot compensate for intrinsic pathway defects observed in hemophilia.^11,18^ These limitations led to the development of the cell-based model, which incorporated cellular interactions into the enzymology of clot formation.^11^

Conceptualized during the expansion of cell biology research, the cell-based model of coagulation describes hemostasis as a dynamic process occurring on cellular surfaces through three overlapping phases, initiation, amplification, and propagation, which together integrate platelet function and coagulation enzymology to achieve effective fibrin formation.^14,19,20^ The initiation phase begins when vascular injury exposes the tissue factor (TF) on subendothelial cells. TF binds to factor VII/VIIa, activating factor X and generating lesser amounts of thrombin on TF-bearing cells.^19^ The amplification phase follows when the initial thrombin produced during initiation activates factor V, factor VIII, and factor XI, enhancing the procoagulant potential of the platelet surface.^19,20^ During the propagation phase, fully activated platelets serve as the main catalytic sites for the formation of the tenase and prothrombinase complexes, leading to a burst of thrombin generation.^19,20^ The newly produced thrombin cleaves fibrinogen into fibrin monomers, which polymerize and are cross-linked by factor XIIIa, forming a stable fibrin network anchored to the platelet plug. The outcome is a localized, tightly regulated clot that seals the vascular injury while limiting systemic activation of coagulation.^12,14,19,21^

Finally, following the clot’s function in wound healing, the fibrinolysis process must solve it. Fibrinolysis, which constitutes tertiary hemostasis, is the proteolytic degradation of the fibrin network that forms the structure of a blood clot, releasing trapped platelets and red blood cells back into the circulation. It begins when a plasminogen activator, mainly tissue plasminogen activator (tPA), converts plasminogen into plasmin, which binds to fibrin and enzymatically cleaves its fibers.^22^ Internal fibrinolysis occurs when tPA is trapped within the clot, driven by pore expansion within the fibrin network, while external fibrinolysis involves therapeutic fibrinolytic administration, such as for myocardial infarction or stroke.^23^ By integrating enzymological and cellular insights, there was also progress in therapy development, resulting in the creation of direct anticoagulants that selectively inhibit the active sites of essential coagulation enzymes.^11^

More recently, an immune-based perspective has emerged, emphasizing the interplay between coagulation and innate immunity, with concepts of immunohemostasis and immunothrombosis becoming particularly evident during COVID-19.^24–26^

As previously outlined, in regular physiological conditions, clot formation is regulated, and once the healing process is complete, the body naturally dissolves the clot. However, in certain situations, thrombosis may develop without an apparent injury, or the body’s mechanisms for clot dissolution may fail.^27^ Thrombosis can be classified into arterial or venous types depending on whether the blood clot forms. Arterial thrombosis typically occurs in high-flux blood environments and is predominantly composed of platelets. Its trigger is often the rupture of atherosclerotic plaques, which exposes subendothelial collagen and von Willebrand factor, leading to rapid platelet activity. This process is the leading cause of ischemic heart disease and stroke, two of the most severe forms of cardiovascular morbidity and mortality.^28^ In contrast, VTE occurs under slow-flow conditions and results in fibrin-rich clots, which trap large numbers of red blood cells. Coagulation factors primarily drive VTE, being strongly associated with Virchow’s triad: venous stasis, endothelial dysfunction, and hypercoagulability. Clinical manifestations include deep vein thrombosis—typically affecting the lower limbs—and pulmonary embolism, a potentially fatal condition caused by clot migration to the lungs.^10^

According classification of Anatomical Therapeutic Chemical Classification System (ATCCS) of the World Health Organization (WHO), antithrombotic agents are a therapeutic group categorized into subgroups as anticoagulants, vitamin K antagonists, heparin group, direct thrombin inhibitors, direct factor Xa inhibitors, platelet aggregation inhibitors, enzymes (fibrinolytics), and other antithrombotic agents.^29^

Antithrombotic agents target key steps in platelet pathways, coagulation cascade, and fibrin degradation and are used for both prevention and treatment of thromboembolic disorders. The first anticoagulants appeared in the early 20th century and are commonly termed traditional agents, including unfractionated heparin (UFH) and warfarin.^30^ However, in recent decades, a new generation of anticoagulants, the direct oral anticoagulants (DOACs), has been developed, specifically designed to target key enzymes in the coagulation cascade—thrombin (factor IIa) and factor Xa—that play central roles in thrombus formation.^31,32^

Besides UFH and the vitamin K antagonist warfarin, traditional agents include low molecular weight heparins (LMWH), like enoxaparin, and the synthetic pentasaccharide fondaparinux (classified as other agents according to ATCCS).^29^ The class of heparins, administered parenterally, enhances antithrombin activity and inhibits thrombin and factor Xa, with LMWH acting more selectively on factor Xa.^33,34^ Oral vitamin K antagonists inhibit the synthesis of vitamin K-dependent clotting factors (II, VII, IX, and X).^35^ Vitamin K functions as a cofactor for the gamma-carboxylation of specific glutamate residues in vitamin K-dependent coagulation factors, generating gamma-carboxyglutamate residues. These residues provide calcium-binding sites that enable the factors to anchor to negatively charged phospholipid surfaces, a step essential for the assembly of coagulation complexes.^36^ Fondaparinux, a synthetic pentasaccharide also administered parenterally, selectively inhibits factor Xa.^35^

Among newer anticoagulants are the DOACs—rivaroxaban, apixaban, and edoxaban—that inhibit factor Xa, thereby preventing the conversion of prothrombin to thrombin. The direct oral thrombin inhibitor dabigatran blocks thrombin (factor IIa), hindering fibrin formation. They represent newer anticoagulants with predictable pharmacokinetics and fewer monitoring requirements.^37^

Antiplatelet agents target platelet activation pathways and are primarily used in arterial thrombosis. Cyclooxygenase-1 inhibitors, such as aspirin, reduce TxA2 synthesis and platelet activation.^38^ P2Y_12_ receptor antagonists, like clopidogrel, prasugrel, and ticagrelor, inhibit ADP-induced platelet aggregation.^39^ Inhibitors of GP IIb/IIIa receptors on platelets, abciximab, eptifibatide, and tirofiban, work by blocking this receptor, preventing fibrinogen from binding, and thus inhibiting platelet aggregation. The administration of these approved GP IIb/IIIa antagonists is intravenous, reflecting their molecular characteristics (proteins and peptide-based) and the need for rapid onset of action in acute clinical settings.^40^

While anticoagulants and antiplatelet agents act primarily by preventing the formation and propagation of thrombi, fibrinolytic agents are used in acute settings to actively dissolve pre-existing clots. Fibrinolytics, including alteplase and tenecteplase, are administered intravenously, acting as recombinant tPAs, converting plasminogen into plasmin to dissolve fibrin-rich thrombin.^41^

Therapeutic strategies for thrombotic disorders aim to restore or maintain blood flow, prevent thrombus propagation, and reduce recurrence with antithrombotic agents. Preventive approaches integrate pharmacological and lifestyle interventions to address endothelial dysfunction. Current management of VTE primarily involves anticoagulation, preferably with DOACs (apixaban, rivaroxaban, dabigatran, and edoxaban) or warfarin when DOACs are unsuitable.^42^ In coronary artery disease (CAD), standard treatment consists of single or dual antiplatelet therapy using aspirin and/or a P2Y_12_ inhibitor (e.g., clopidogrel, prasugrel, and ticagrelor), with low-dose rivaroxaban as an option, individualized according to ischemic and bleeding risks.^43^ For stroke prevention, the recommendation is DOACs or warfarin for atrial fibrillation and antiplatelet therapy for noncardioembolic stroke, emphasizing short-term dual therapy in minor ischemic events and comprehensive risk-factor control.^44^ Optimizing patient outcomes often requires individualized treatment regimens, balancing efficacy with risk complications.

Given the high morbidity and mortality associated with thrombosis, there is growing interest in exploring alternative materials and administration routes that can improve patient outcomes.^45^ The skin, given its complex multilayered structure and barrier properties, plays a critical role in transdermal drug delivery, influencing the permeation flux and rate of drugs into systemic circulation.^46^ As seen in Figure 2, the skin is a stratified tissue composed of the epidermis, dermis, and hypodermis (or subcutaneous tissue). As the outer layer, the epidermis serves primarily as a protective barrier against external aggressions. It has further subcategorizations based on the degree of keratinization and other intrinsic characteristics, divided into the stratum basale, stratum spinosum, stratum granulosum, stratum lucidum, and stratum corneum. The first three layers are collectively referred to as the viable epidermis. Beneath the epidermis lies the dermis, closely connected through the dermoepidermal junction. This layer contains the skin’s appendages, including hair follicles, sweat and sebaceous glands, blood and lymphatic vessels, and neural components. It is responsible for skin’s structural support and elasticity.^47–49^

**Figure 2:**
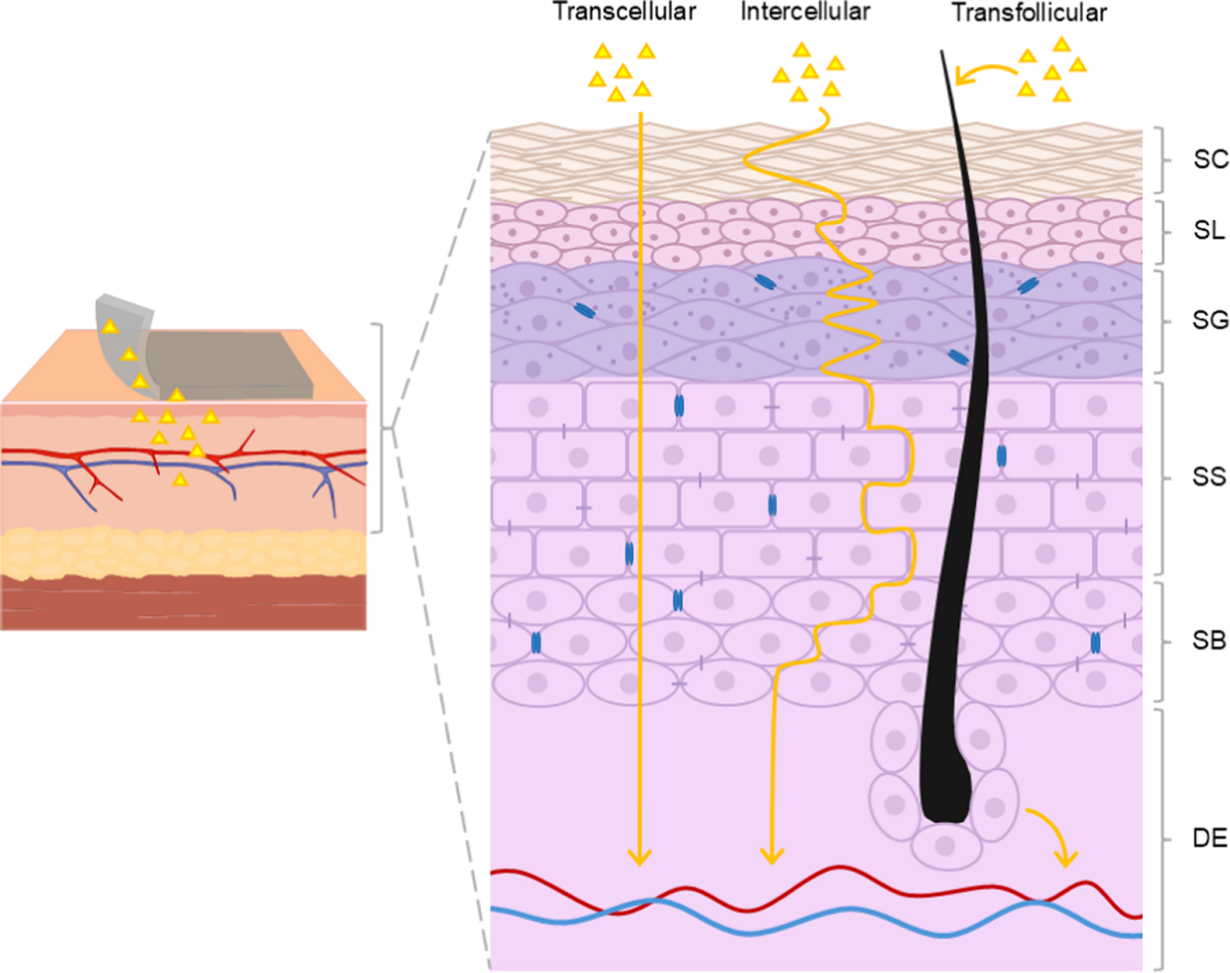
Schematic representation of the different pathways for drug permeation across skin layers. SC: stratum corneum; SL: stratum lucidum; SG: stratum granulosum; SS: stratum spinosum; SB: stratum basale; DE: dermis.

Drug penetration through the skin occurs mainly via three pathways: transcellular, intercellular, and transfollicular. In the transcellular route, molecules pass through the cell cytoplasm, alternating between lipophilic and hydrophilic regions within keratinocytes; this is a short path, but a challenging one due to multiple membrane passings. The paracellular route consists of drugs moving through the lipidic intercellular spaces, favoring lipophilic compounds but limiting hydrophilic ones due to the lipid barrier and compact stratum corneum. The transfollicular route, in turn, allows penetration through skin appendages like hair follicles and glands, offering an alternative entry pathway but is extremely limited because of the small absorption area.^49,50^

Despite the skin’s highly selective barrier, its vast surface area and the painless convenience of transdermal dosing have propelled transdermal drug delivery into a rapidly expanding modality, promising better bioavailability, adherence, and efficacy with fewer side effects.^46,51^ Although superficial/topical antithrombotic formulations have been explored over recent years, especially for wound healing, transdermal antithrombotic delivery remains a challenge.^52–54^ Even so, transdermal drug delivery systems (TDDS) are attractive options for drugs with limited oral bioavailability, significant presystemic metabolism, rapid gastrointestinal degradation, or poor aqueous solubility. By bypassing first-pass metabolism, TDDS can stabilize plasma concentrations, facilitate on–off control, assist bedridden patients, and often allow lower daily therapeutic doses than oral administration, thereby mitigating systemic adverse effects such as bleeding and gastric irritation. As a noninvasive and less painful alternative to injections, TDDS may also improve patient adherence and reduce the need for healthcare professionals’ involvement in drug administration.^55,56^ In this context, this perspective aims to provide an overview of recent advances in transdermal antithrombotic therapy, exploring studies on transdermal technologies and assessing the benefits and challenges in developing safe and efficacy antithrombotic treatment using this route of administration.

## Results and Discussion

### Overview of the Included Studies

As illustrated in Figure 3, there were 976 records using all different search combinations, resulting in 383 nonduplicated articles. There was a first screening of these titles and abstracts, and it led to 67 papers, excluding terms such as wound healing, scar treatment, and tissue engineering, as these suggest a topical effect, rather than transdermal and systemic anticoagulant action. After the search, there was a full-text review of relevant articles, which provided 25 viable articles and 42 exclusions based on eligibility criteria: different administration routes (5), nonantithrombotic treatments, e.g., wound healing (17), and other factors (20). This last category includes review articles and case reports (10), studies whose focus was not on transdermal antithrombotic therapy but instead used active pharmaceutical ingredients (APIs) as models to validate innovative technologies or methodologies (7), and articles with an impact factor below 1.5 or without full-text access (3).

**Figure 3:**
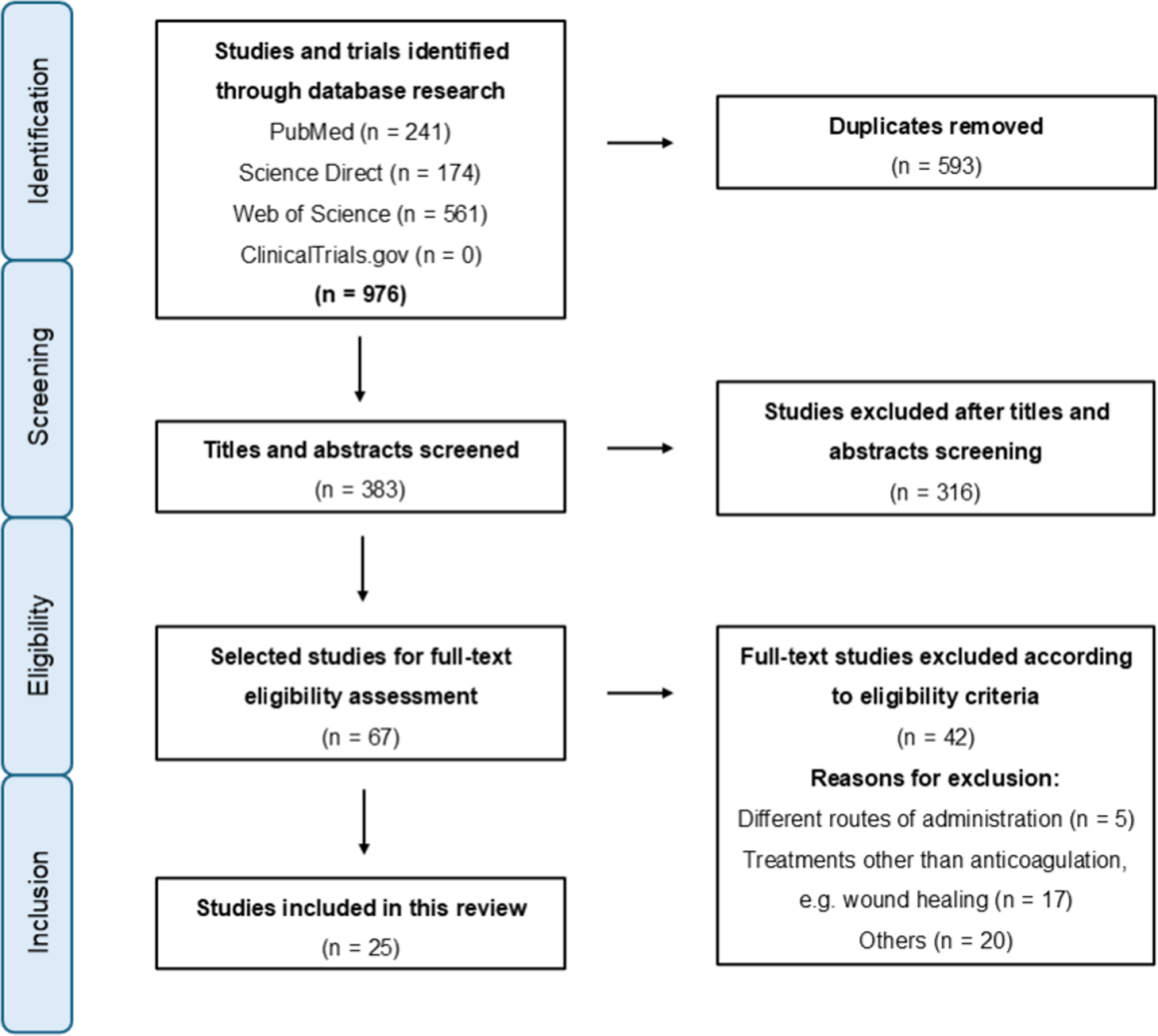
Studies selection based on the PRISMA flowchart.

Among the selected articles, Table 1 shows 25 research studies. Within the TDDS technology studies, 10 focused on heparins, 9 on acetylsalicylic acid (ASA), 3 on DOACs, and 3 on other substances such as fucoidan and hirudin. For heparins, 6 studies utilized polymeric microneedles, while 4 employed polymeric patches, microemulsions, or gel/solution formulations. In the case of ASA, the identified studies included hydrogels (3), polymeric microneedles (4), and polymeric films (2). For DOACs, the research included studies on rivaroxaban and apixaban using microemulsion-based hydrogels (1), ultrafine oil/water (O/W) nanoemulsions (1), and ethosome gels (1). For fucoidan and hirudin, microneedles were the primary technology researched, consisting of 3 articles.

**Table 1:** Summary of Reviewed Articles: API and Their Transdermal Drug Delivery System (TDDS) Technologies

API	API content	TDDS	technique	in vitro*/*ex vivo*/*in vivo studies	authors/reference
Heparin	250 IU	polymeric microneedle	vacuum micromolding	in vitro	Arshad et al.^57^
				• drug release ex vivo	
				• skin insertion	
				• drug permeation in vivo	
				• pharmacodynamics	
				• histology	
Heparin	10 mg	polymeric patch	solvent casting	ex vivo	Patel et al.^58^
				• drug permeation	
Heparin	1% w/v	hydrogel microneedle	digital-light processing-based 3D printing	in vitro	Sun et al.^59^
				• cytotoxicity	
				• bioactivity	
Heparin	0.3% w/v	gel and solution	ablative laser pretreatment of skin	in vitro	Vora et al.^60^
				• drug release ex vivo	
				• drug permeation	
Heparin	2268 IU/kg	ultrarapid polymeric microneedle	mold-based polymerization under ultraviolet (UV)	in vitro	You et al.^61^
				• drug release in vivo	
				• pharmacodynamics	
				• safety (skin irritation and tolerability)	
Heparin	not reported	sponge spicules microneedle	low frequency sonophoresis combined with microneedle	in vitro	Zhai et al.^62^
				• drug release ex vivo	
				• drug permeation in vivo	
				• pharmacodynamics	
				• safety (skin irritation)	
Heparin	200 IU/kg	hydrogel microneedle	cross-linked hydrogel loaded in microneedles mold	in vitro	Zhang et al. (2017)^63^
				• drug release	
				• bioactivity in vivo	
				• pharmacodynamics	
				• drug release (fluorescence)	
Heparin	4000 IU/mL	methacrylate gelatin-composed ice microneedle	polymer casting and freezing	in vitro	Zhang et al. (2021)^64^
				• drug release ex vivo	
				• skin insertion in vivo	
				• pharmacodynamics	
enoxaparin	not reported	liposomal gel	authorial method	in vitro	Jain et al.^65^
				• drug release	
				• bioactivity	
				• biocompatibility (hemolysis assay)	
enoxaparin	100 mg/mL	Microemulsion	titration	ex vivo	Alkrad et al.^66^
				• drug permeation in vivo	
				• pharmacodynamics	
ASA	100 mg	pullulan hydrogel	solvent casting	in vitro	Kongmee et al.^67^
				• drug release	
ASA	200 mg	biopolymeric microneedle	AdminPatch Microneedle Arrays	in vitro	Olatunji et al.^68^
				• drug release ex vivo	
				• skin insertion	
ASA	9.52 ± 0.46 mg	hydrogel	solution casting	in vitro	Pairatwachapun et al.^69^
				• drug release	
ASA	75, 150, 300 mg/mL	Nanorods hydrogel	chemical precipitation/Microwave-assisted synthesis	in vitro	Radwan-Pragłowska et al.^70^
				• drug release	
				• cytotoxicity	
ASA	80 mg	microneedle-assisted transfersome	injection molding	in vitro	Rahbari et al.^71^
				• drug release	
				• cytotoxicity ex vivo	
				• drug permeation	
ionic liquid lidocaine/ASA	20, 40, 60 μg/mL	Gelatin/polymeric composite film	Freeze–thaw	in vitro	Maneewattanapinyo et al.^72^
				• drug release ex vivo	
				• drug permeation	
Ionic liquid lidocaine/ASA	4.5 g	polymeric film	solvent casting	in vitro	Suksaeree et al.^73^
				• drug release	
ASA	2.5 mg per patch	dissolving polymeric microneedle	microneedles molding with aspirin concentrated in the tip	in vitro	Wang et al. (2023a)^74^
				• drug release	
				• dissolution ex vivo	
				• skin insertion in vivo	
				• pharmacodynamics	
				• pharmacokinetics	
				• dissolution	
				• safety (skin irritation)	
ASA	6 mg per patch	dissolving polymeric microneedle	microneedles molding	in vivo	Wang et al. (2023b)^75^
				• pharmacokinetics	
				• dissolution	
				• safety (skin irritation)	
Rivaroxaban	0.3–0.4 mg/g	microemulsion-based hydrogel	spontaneous emulsification	in vitro	Araújo et al.^76^
				• drug release	
				• cytotoxicity ex vivo	
				• drug permeation	
Apixaban	5 mg	Ultrafine O/W Nanoemulsion	titration	in vitro	Abdulbaqi et al.^77^
				• drug release ex vivo	
				• drug permeation	
Apixaban	10 mg/mL	Ethosome gel	thin-film hydration	in vitro	El-Shenawy et al.^52^
				• drug release ex vivo	
				• drug permeation in vivo	
				• pharmacokinetics	
				• safety (skin irritation)	
Fucoidan	0.48 mg	dissolving gelatin/polymeric microneedle	Micromolding	ex vivo	Stephanie et al.^78^
				• drug permeation in vivo	
				• pharmacokinetics	
Hirudin	1–20 mg/mL	dissolving polymeric microneedle	3D-printed array modelMicroneedles fabrication by casting	in vitro	Wu et al.^79^
				• drug release ex vivo	
				• Skin insertion in vivo	
				• pharmacodynamics	
				• pharmacokinetics	
				• safety (skin irritation)	
				• Histology	
recombinant hirudin	25, 50, 100 μg	dissolving polymeric microneedle	mold casting	in vitro	Men et al.^80^
				• drug release ex vivo	
				• skin insertion in vivo	
				• pharmacodynamics	
				• pharmacokinetics	
				• dissolution	
				• safety (skin irritation, skin recovery)	

### Promising Systems for Transdermal Delivery of Antithrombotic Agents

TDDSs offer a promising alternative to oral administration by bypassing gastrointestinal and hepatic degradation, thereby reducing side effects, enhancing bioavailability, and improving patient compliance.^81,82^ However, the stratum corneum poses significant challenges on the transdermal delivery of molecules and drugs due to its barrier properties, particularly limiting the permeation of substances with higher molecular weight than 500 Da and some lipophilic compounds with molecular weights below 350 Da.^71^ Examples of antithrombotic agents with such characteristic and limited skin penetration include heparin with 15 to 18 kDa and fucoidan from 10 to 300 kDa, both of which exhibit limited skin penetration.^68,78^ Thus, numerous transdermal drug delivery techniques have been developed to overcome this barrier, such as microneedles, hydrogels, iontophoresis, topical patches, and nanometric systems, among others (Figure 4).^62,71^

**Figure 4:**
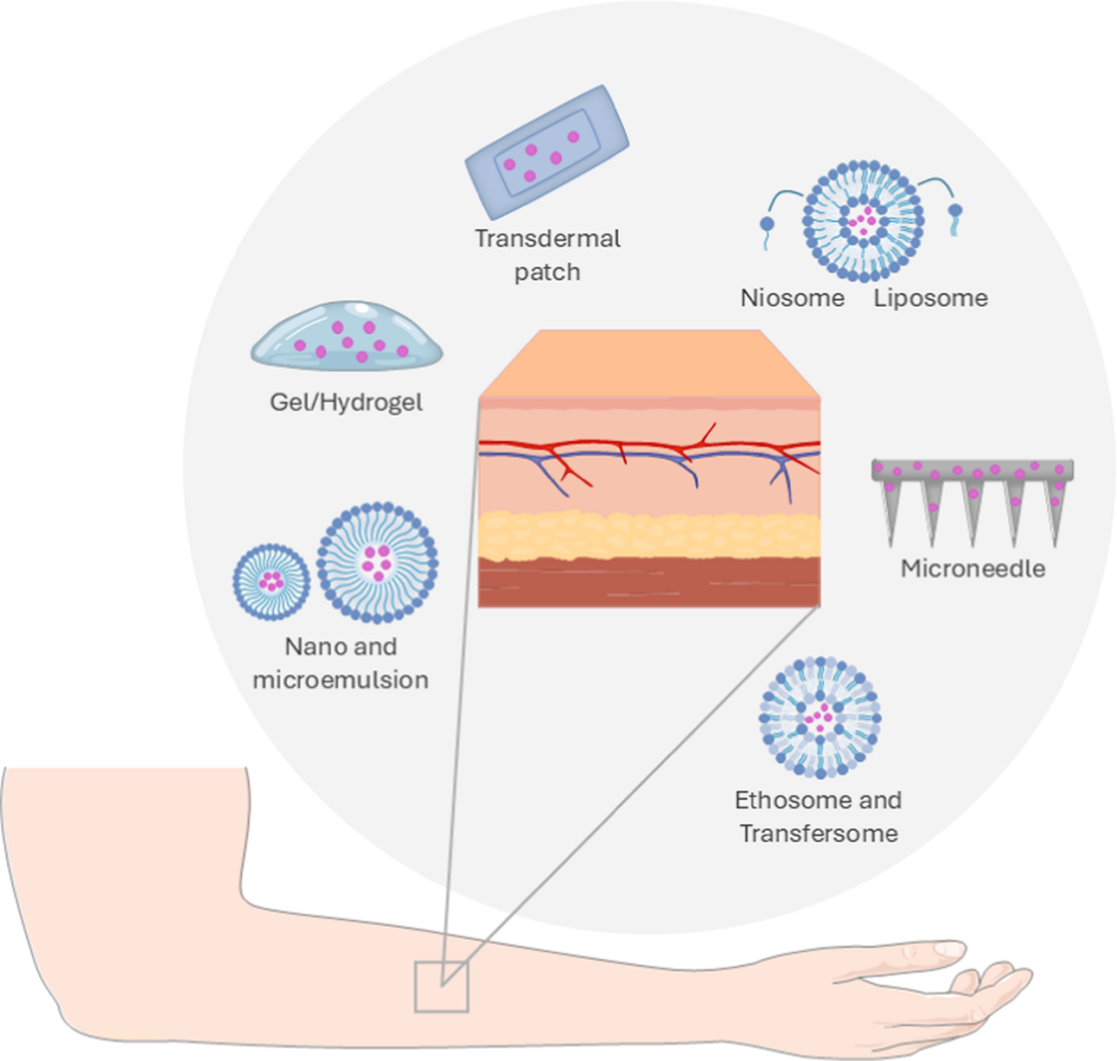
Demonstration of skin layers and different innovative TDDSs used to enhance antithrombotic API permeation.

Microneedles have emerged as a minimally invasive approach to deliver antithrombotic drugs across the skin.^57,74,79,80^ They are micrometer-sized vertical projections with sharp tips erected from a flat substrate and designed to be long enough to pierce the upper layer of the skin, the stratum corneum, and reach into the viable epidermis. However, they are sufficiently short to avoid penetrating the deeper dermal layer, where pain receptors and blood vessels are located. Therefore, they function as painless mechanical penetration enhancers. After the insertion of the solid microneedles, drug formulations can be applied over the resulting micropores on the skin surface, facilitating the diffusion of therapeutic agents through the skin layers and enabling either local or systemic effects. Although their mechanism of action relies on the transient creation of microchannels, these pores tend to close rapidly due to the skin’s natural elastic retraction.^59,68,71^ Various materials have been used for the fabrication of microneedles, including silicon, metal, ceramic, glass, sugar, and polymer.^83–88^ Polymers are the most used materials for the fabrication of microneedles due to their biocompatibility, low toxicity, and drug delivery properties. Additionally, it is possible to avoid denaturation or destabilization of a drug by enclosing it in a polymer matrix.^57,71,80^

The combination of microneedles with nanocarrier-based drug delivery systems has been explored, such as transfersomes to enhance antithrombotic drug permeation, enabling controlled release and improving both local and systemic delivery.^71^ Transfersomes are highly deformable lipid-based vesicles composed mainly of phospholipids and edge activators such as surfactants. Features of these carriers are increased elasticity and deformability of the lipid bilayer, as well as the osmotic gradient around the skin. This flexibility enables them to pass through narrow skin channels 10 times smaller than their own diameter without drug leakage, resulting in improved permeation through the stratum corneum into deeper skin layers, higher entrapment efficiency of the API, and controlled release at the target site. Thus, transfersomes improve transdermal drug delivery by two distinct mechanisms: first, as drug carriers, they diffuse into the stratum corneum and transport encapsulated drugs into deeper skin layers; second, they enhance permeation by disrupting the lipid structure of the stratum corneum, modifying the intercellular lipid bilayer, fusing with it, and releasing the drug.^71,89,90^ Despite their advantages, transfersomal delivery systems tend to be chemically unstable, highly dependent on phospholipid purity, and relatively costly.^91^

Polymeric systems such as hydrogels have also been investigated for transdermal antithrombotic drug delivery. Hydrogels are biocompatible, nontoxic, and highly hydrated polymeric networks that resemble biological tissues. They have several characteristics, including the ability to swell, responsiveness to pH, temperature, electric fields, and ionic strength, making them attractive platforms for controlled drug delivery. However, slow drug release kinetics and reduced capacity to transport drugs with high molecular sizes limit their applications in transdermal delivery.^92,93^ Since the electronic conductivity of a hydrogel is generally low, iontophoresis, a method that applies a low-level electric current (0.5 mA/cm^2^ or less), has been considered to overcome these limitations. It has been employed to facilitate the movement of drug ions across the membrane and to enhance the skin permeation and the release rate of drugs that have poor absorption or permeation profile through the skin by electrophoresis force. Recently, a conductive polymer combined with a hydrogel has attracted attention as an electroactive hydrogel that is capable of chemical or physical transformations in response to electrical potential.^67,69^

Poloxamer-based gels have shown potential as carriers for the transdermal delivery of antithrombotic drugs, particularly when combined with laser pretreatment of the skin. Poloxamers are thermoresponsive triblock copolymers that remain in a liquid state at room temperature and undergo sol–gel transition at skin or body temperature, allowing for sustained drug release at the site of application. Laser-assisted delivery, especially using ablative lasers that remove the stratum corneum, has been shown to significantly enhance the permeation of hydrophilic macromolecules such as heparin. While nonablative lasers facilitate the delivery of small molecules by increasing skin permeability without disrupting the barrier layer, they are less effective for larger hydrophilic compounds.^60^

Beyond gel-based systems, transdermal patches have also demonstrated significant potential for sustained antithrombotic drug release, offering the additional advantage of reducing the need for frequent applications compared to topical gels.^94^ Transdermal patches are structured as multilayered polymeric laminates, in which the API is incorporated either into a drug reservoir or a drug–polymer matrix positioned between two polymeric layers: an outer, impermeable backing layer that prevents drug loss to the external environment and an inner polymeric layer that interfaces with the skin and may function as an adhesive or a release-controlling membrane. Modified transdermal patches may also be produced with one or more layers composed of nanofibers and polymeric films. The drug release profile can be modified using hydrophilic or hydrophobic polymers. In addition, the use of permeation enhancers such as oleic acid and isopropyl myristate improved drug permeation across the skin.^58,72,95,96^

Nanometric systems, including liposomes, have also been explored to improve antithrombotic drug permeation and achieve sustained and controlled release profiles. Liposomes are lipid-based vesicles with amphiphilic properties, such as the epidermis. This property facilitates their interaction with the skin, enhances penetration through the epidermal barrier, and provides a sustained and controlled delivery.^65,94,97^ Propylene glycol liposomes incorporated into gel formulations have shown promising results for topical anticoagulant therapy. This system has been investigated for the effective delivery of LMWH into and across the skin with the aim of improving its bioavailability. Propylene glycol acts as a penetration enhancer and, when combined with other enhancers, exerts a synergistic effect on skin permeability.

Microemulsions are thermodynamically stable, isotropic, and clear nanosystems that spontaneously exist as water-in-oil or oil-in-water dispersions. They are composed of a hydrophilic phase, a lipophilic phase, surfactants, and cosurfactants with a nanometric droplet size usually below 100 nm. Microemulsions are suitable for transdermal antithrombotic drug delivery, as they can enhance the solubilization of poorly water-soluble drugs, producing higher drug loading and increasing the concentration gradient across the skin, which serves as a major driving force for drug permeation. Moreover, some ingredients used in microemulsion formulations can behave as permeation enhancers that can overcome the barrier functions of the skin. These compounds may already exhibit enhanced skin permeability due to their chemical properties.^66,76^ Nanoemulsion dispersion can also be a promising tool for transdermal drug administration. They display uniform distribution with droplet size ranging from 20–200 nm, which allows high drug flux and penetration through the skin’s intracellular lipophilic pathways. Droplets smaller than 20 nm can easily permeate the skin and contribute to the formation of a drug depot within the stratum corneum and epidermis.^77^

Antithrombotic drugs can also pass through the nasal epithelium by either paracellular or transcellular pathways; however, it is possible to observe bleeding risks. The transportation of lipophilic drugs is primarily transcellular, demonstrating efficient absorption and high bioavailability. In contrast, the transportation of hydrophilic drugs (Biopharmaceutical Classification System Class III, high solubility and low permeability) is through the paracellular route, where absorption is often incomplete due to the presence of tight junctions between epithelial cells. To increase nasal membrane permeability, there were investigations of several nanocarrier systems such as liposomes, niosomes, and ethosomes, as they have been employed for the delivery of both hydrophilic and lipophilic drugs, offering benefits such as controlled release, protection of the encapsulated drug, and targeted delivery.^52^ Niosomes are self-assembled vesicles, ranging from 300 to 500 nm in size, composed mainly of nonionic surfactants in combination with cholesterol or other lipids.^98^ Their structure resembles that of liposomes, with polar head groups oriented toward the aqueous core and hydrophobic tails forming the bilayer, which faces the external environment.^56^ Although like liposomes, niosomes are generally more stable due to the materials used in their preparation, providing several advantages such as enhanced penetration capability, lower production cost, easier storage, and reduced toxicity.^98,99^ Furthermore, nonionic surfactants contribute to increased membrane permeability and fluidity, and their ability to enhance solubility has been widely applied to improve the bioavailability of poorly water-soluble drugs.^100^ Ethosomes, in particular, are soft, malleable vesicles composed of phospholipids and a high concentration of alcohol, which enhances membrane permeability.^52^ Their size ranges from approximately 30 nm to several microns. Compared with liposomes, ethosomes are more effective in delivering drugs through the skin and via the intranasal route, in terms of both quantity and depth of penetration, while also providing a higher cost-to-benefit ratio. Due to their unique structure, ethosomes can encapsulate and deliver highly lipophilic as well as cationic drugs through the skin. Once in contact with cells, they can penetrate the cellular membrane and release the entrapped molecules intracellularly.^52,99^

The main TDDSs for antithrombotic drugs are listed in Figure 4.

### Antithrombotic Drugs Used in Transdermal Treatment

#### Heparins

Heparin is an anticoagulant listed as an essential medicine by WHO.^101^ Heparins are classified into two main forms: UFH and LMWH, such as enoxaparin, and are commercially available in injectable formulations for systemic treatment, administered either intravenously or subcutaneously.^102,103^ UFH, with its higher molecular weight (12–16 kDa) and longer polysaccharide chains, is able to form a ternary complex with antithrombin and thrombin, thereby effectively inhibiting factor IIa (thrombin) and factor Xa, while also showing weaker inhibitory effects on factors IXa, XIa, and XIIa.^33,34^ LMWH has a lower molecular weight (3.8 to 5 kDa) and selectively inhibits factor Xa, providing more predictable anticoagulation with fewer side effects.^33^ Due to their low stability in acidic pH and rapid metabolism in the gastrointestinal tract, heparins show low oral bioavailability, limiting their use to injectable pharmaceutical forms, which are primarily administered in hospitals.^66,104^ Besides low oral bioavailability, UFH injectable forms also present a short half-life and frequent dosing, often leading to systemic side effects, listed in Figure 5, and poor patient compliance.^105–107^ Transdermal drug delivery offers a promising alternative to conventional injectable routes. Its noninvasive, convenient, and painless characteristics enhance patient compliance, while features such as controlled and prolonged release, reduced frequency of administration, easy discontinuation, and the elimination of healthcare professionals’ involvement further support its advantages over a subcutaneous injection. Additionally, by lowering the risk of disease transmission and improving drug bioavailability, transdermal delivery represents a viable option for patients in need of noninvasive therapeutic approaches.^81,82^ Aiming to overcome these issues, several transdermal formulations have been proposed to deliver heparin macromolecules using technologies such as microneedles and nonmicroneedle formulations (microemulsion, patches, etc.)^57–66^

**Figure 5:**
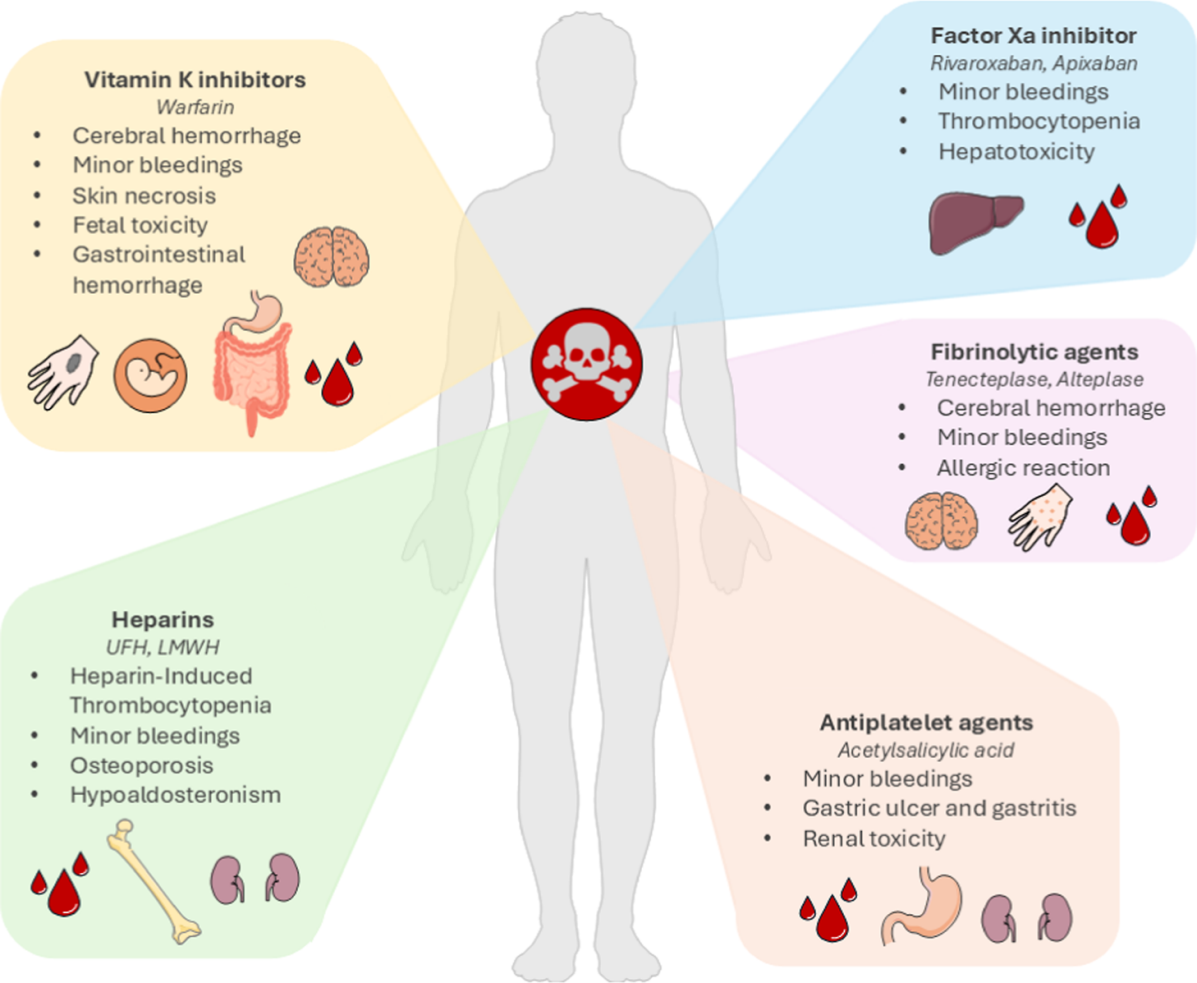
Visual summary of antithrombotic-associated risks, showing affected organs and potential complications. Minor bleedings are usually general and manageable without interventions (e.g., bruises, nose bleeding, and ecchymosis), while specific bleedings affect internal organs (e.g., gastrointestinal and intracranial hemorrhage) and require urgent medical attention with antithrombotic therapy adjustment.

Sun et al., Zhang et al. (2017), You et al., Zhang et al. (2021), and Arshad et al. each proposed microneedle systems for transdermal delivery of heparin, directly targeting the bloodstream to optimize anticoagulant administration and therapeutic efficacy.^57,59,61,63,64^ Sun et al. introduced a 3D-printed microneedle array inspired by succulent plants, designed to allow controlled and sustained heparin release via an external near-infrared (NIR) stimulus.^59^ This approach enhances control over release rate and promotes prolonged skin adhesion, which is beneficial for consistent therapeutic effects.

Zhang et al. (2017), Arshad et al., and You et al. further developed polymeric microneedles.^57,61,63^ Arshad et al. prepared self-applying microneedle patches composed of poly­(vinyl alcohol) (PVA).^57^ The purpose of this biocompatible and biodegradable PVA formulation was to offer microneedles with a proper endurance attribute, enabling an effective and painless heparin skin delivery, thereby improving patient adherence. Results from these studies indicate that such patches could promote effective heparin release over time. Using polymer-based microneedles responsive to thrombin, Zhang et al. (2017)^63^ proposed a “smart” system that enables heparin release only when thrombin is present. The thrombin-responsive matrix was prepared by polymerizing a heparin-conjugated hyaluronic acid with a cleavable peptide under ultraviolet (UV) light. In the presence of activated thrombin, the short peptide is specifically recognized and cleaved, enabling targeted release of heparin.^108^ This release mechanism was designed to work as a feedback system, aiming to prevent blood clot formation while avoiding unnecessary drug exposure and mitigating risks associated with continuous heparin, like bleeding. Differently, providing a controlled but immediate heparin release, the work of You et al. reported ultrarapid-release microneedles with CO_2_-generating agents.^61^ Biodegradable solid microneedles, embedded with effervescent agents, offered rapid heparin release for acute cases requiring immediate anticoagulation. The intention of this potential product is to balance controlled rapidity with efficacy in high-risk settings, although it is intended for short-term rather than sustained release. Exploring soft and frozen materials, Zhang et al. (2021) proposed a microneedle patch technology using an ice-based structure instead of conventional solid polymeric materials.^64^ These ice microneedles, prepared from water-containing biomaterials such as Matrigel, methacrylated gelatin, sodium alginate, and other soft hydrogels, achieved over 90% penetration efficiency and successfully delivered actives such as heparin, erythropoietin, and *Bacillus subtilis*, demonstrating their potential for transdermal applications. Because the manufacturing process occurs under mild, low-temperature conditions, the authors highlighted that this approach is particularly advantageous for the delivery of thermolabile actives, including proteins, peptides, and even living microorganisms, whose bioactivity can be preserved throughout processing. This innovation removes the dependence on conventional polymeric scaffolds in microneedles, which impose limitations related to processing temperature, drug compatibility, mechanical strength control, and the need for chemical cross-linking or drying steps.

Although less prevalent in the bibliography reviewed, nonmicroneedle technologies remain under investigation for heparin transdermal delivery. Patel et al. explored transdermal absorption of heparin using polymeric patches produced by the solvent evaporation method.^58^ Their study demonstrated that a formulation containing 10% oleic acid and 10% isopropyl myristate performed with a 1.369-fold enhancement in drug permeation. Zhai et al. and Vora et al. explored physical methods to assess transdermal delivery of heparin.^60,62^ Vora et al. used an ablative laser technology to pretreat the skin for delivering 0.3% w/v heparin-loaded poloxamer gel and solution.^60^ Comparative in vitro permeation studies using static Franz diffusion cells revealed no passive delivery of heparin; however, laser-assisted delivery of heparin from the solution (26.07 ± 1.82 μg/cm^2^) outperformed the delivery from the gel (11.28 ± 5.32 μg/cm^2^). Even though there were better results with the heparin solution, gel formulation may support prolonged drug release, functioning as a maintenance dose skin to continuous intravenous infusion.^60^ Similarly, Zhai et al. demonstrated that low frequency sonophoresis, combined with sponge *Haliclona* sp. spicules, enhanced transdermal drug delivery.^62^ The topical application of the combination was able to increase in vitro skin absorption of fluorescent dextrans, a model compound for low-molecular-weight heparin. According to the authors, this enhancement could be attributed to the synergistic effects of long-lasting nanochannels created by sponge *Haliclona* sp. spicules and the disruption of stratum corneum lipids caused by low-frequency sonophoresis shock waves, which improved cavitation homogeneity.

As observed in UFH studies, there were similar efforts by LMWH to develop alternatives to injectable administration. Alkrad et al. evaluated the pharmacokinetics of enoxaparin-loaded microemulsions administered via oral and transdermal routes in rats, comparing them with a commercially available subcutaneous formulation.^66^ The findings revealed no significant difference in bioavailability between the transdermal microemulsion and subcutaneous formulation. However, the oral administration of microemulsions exhibited a significantly lower bioavailability. In the same manner, Jain et al. developed propylene glycol liposomes encapsulating enoxaparin with high encapsulation efficacy, sustained drug release, and effective anticoagulant activity.^65^

Transdermal formulations with heparin have attracted significant interest in recent research, as it remains a commonly used drug that is still available only in injectable form. Microneedles appear to be particularly interesting once they potentially enable skin penetration of large and high charge density molecules such as heparin. Although there have been observed advances in heparin transdermal delivery systems, distinct challenges and gaps still need further studies. Future efforts might be employed to address challenges such as achieving stable and/or prolonged heparin, manufacturing, and logistical complexities—possibly being a scalability concern—and limitation in achieving high drug-loading capacities.^45,57,59,61,63,64^

#### Acetylsalicylic Acid

Acetylsalicylic acid (ASA), or aspirin, is an antiplatelet drug that irreversibly inhibits COX-1 and COX-2, the enzymes that transform arachidonic acid into TxA2, which is a vasoconstrictor and agonist of platelet aggregation.^109^ Because aspirin has a short half-life in plasma concentrations and given the acidic nature of aspirin, taken orally may cause digestive issues and require repeated dosages to achieve long-term platelet aggregation control.^110^

Recent studies have highlighted innovations in transdermal delivery systems for ASA. A diverse approach was observed in the literature screened, using hydrogels, hydrogel patches, polymeric films, and microneedles.^67–75^ All transdermal approaches should allow for bioavailability improvement and minimize the gastrointestinal side effects associated with oral ASA.

Studies of Rahbari et al. and Olatunji et al. explored the strengths of microneedles to bypass the stratum corneum and deliver therapeutic agents directly into deeper layers.^68,71^ In this approach, microneedles assisted the skin perforation to facilitate drug penetration rather than manufacturing microneedles with ASA loaded. Rahbari et al. employed transfersomes—highly deformable lipid vesicles—to encapsulate ASA and enhance its delivery through skin microchannels created by solid silicon and polycarbonate microneedles.^71^ According to the authors, results demonstrated that transfersomes could solve solubility issues of low-water-soluble drugs and enable their slow and controlled release. With a similar idea, Olatunji et al. utilized solid metal microneedles to facilitate ASA penetration from biopolymer films.^68^ The biocompatibility and natural adhesive properties of fish scale biopolymers should allow prolonged ASA release and reduce the need for frequent patch reapplication.

Not only as an assistant, but dissolvable polymeric microneedles were explored in both studies of Wang et al.^74,75^ Polymers like PVA and polyvinylpyrrolidone (PVP) could deliver ASA directly into systemic circulation. This approach maintains effective plasma levels with a reduced dosage, enhancing antiplatelet efficacy and minimizing adverse effects. Additionally, microneedles showed mild skin irritation, which resolved within 24 h, demonstrating high biocompatibility and patient tolerance.^75^ An additional study of Wang et al. (2023a) investigated an innovative approach of embedding high-dose ASA microcrystals at the tips of dissolvable microneedles^74^ The microcrystalline form enhances ASA stability during manufacturing by limiting solvent exposure, which prevents hydrolysis. This method allowed for a gradual, sustained release of ASA, producing stable plasma concentrations with minimal skin irritation, making it particularly promising for chronic therapeutic applications.

Differently, formulations of hydrogel and hydrogel patch as transdermal drug delivery were suggested by Pairatwachapun et al., Radwan-Pragłowska et al., and Kongmee et al.^67,69,70^ Radwan-Pragłowska et al.’s study results showed that a transdermal delivery system carrying ASA made by cross-linked chitosan using azelaic acid, followed by doping with ZnO, was able to promote a sustained release profile, which enhances drug bioavailability while minimizing cytotoxicity.^70^ Kongmee et al. developed an electrically stimulated pullulan hydrogel patch for ASA release. According to research, the hydrogel patch offered a platform for drug delivery, enhancing controlled release through iontophoresis.^67^ Other authors who suggested using external stimuli to enhance skin drug delivery were Pairatwachapun et al.^69^ An electric field was used to facilitate ASA release from polythiophene/carrageenan hydrogel preparation. The conductive polythiophene polymer enhances drug release upon electrical stimulation, enabling precise control over the release rate and offering potential benefits for tailored dosing based on patient needs.

Contributing to ASA advancements in transdermal delivery, Suksaeree et al. developed solvent-cast polymeric films composed of pectin and Eudragit NE 30D (ethyl acrylate and methyl methacrylate copolymer), and Maneewattanapinyo et al. developed gelatin and PVA composite film, which were specifically designed for the delivery of ionic liquid drugs such as lidocaine/aspirin.^72,73^ Suksaeree et al. films exhibited good physicochemical stability, remained amorphous after drug incorporation, and presented rough surfaces due to drug distribution. drug entrapment efficiency varied according to polymer composition, ranging from 103.09% (lidocaine) and 98.95% (aspirin) to 55.47% and 53.76%, respectively. In vitro release followed Higuchi’s diffusion model, governed by diffusion, demonstrating controlled release, with cumulative drug release after 12 h reaching 107.85% (lidocaine) and 75.74% (aspirin). Consequently, it was observed that increasing the fraction of Eudragit NE 30D a hydrophobic and water-insoluble polymer, resulted in a reduced drug release rate.^73^ Maneewattanapinyo et al. developed gelatin/PVA composite films by a freeze–thaw procedure, which was also designed for lidocaine/aspirin delivery. These formulations showed uniform drug distribution with loading efficiencies of 101.46% (lidocaine) and 95.15% (aspirin). In vitro studies confirmed sustained release and skin permeation through porcine skin, with stability testing demonstrating that drug release was best preserved when patches were stored at 4 °C, while higher storage temperatures reduced release efficiency.^72^ As a summary of positive outcomes, both films exhibited structural integrity and biocompatibility, enabling effective transdermal drug delivery, while providing advantages in terms of stability and controlled drug release.

The reviewed literature showed varied approaches for transdermal aspirin delivery. Stability issues are a common challenge. ASA’s tendency to hydrolyze makes it difficult to maintain effective concentrations over time, especially under environmental exposure during storage and use.^74,75^ Rahbari et al. and Wang et al. (2023b) addressed this by employing encapsulation within transfersomes and microcrystal formulation, respectively.^71,75^ Microneedle-assisted technologies have shown promise in enhancing skin permeability and bioavailability, but variability in penetration depth and drug release rates may be a concern, since precise application techniques are necessary. Additionally, hydrogels and polymeric films, while biocompatible and versatile, may face long-term efficacy issues in controlled-release applications. Future work focusing on optimizing stability, consistent bioavailability, and convenient use might be interesting initiatives to support additional advances in ASA formulations.

#### Direct Oral Anticoagulants

Direct oral anticoagulants (DOACs) directly target specific enzymes within the coagulation cascade, such as factor Xa or thrombin, offering significant advantages. As shown in Figure 1, new oral anticoagulants function as factor Xa inhibitors, including rivaroxaban and apixaban, which inhibit the conversion of prothrombin to thrombin.^111^ This mechanism enables predictable dosing, eliminating routine monitoring, thus optimizing treatment and improving patient adherence.^112,113^ Although the recommendation is DOACs as first-line treatments for conditions like atrial fibrillation and VTE, several important issues remain unresolved.^114–116^ As shown in Figure 5, side effects include an increased risk of gastrointestinal bleeding, potential for elevated bleeding risk due to liver dysfunction and interactions with inducers or inhibitors of P-glycoprotein and cytochrome P450 3A4, and concerns related to low bioavailability in certain cases.^117^

Although these drugs present predictable pharmacokinetics and pharmacodynamics with less frequent monitoring requirements, there are circumstances in which monitoring becomes necessary, such as bleeding, within 24 h prior to invasive procedures after DOAC ingestion, recurrent thrombosis after treatment, overdose, and renal or hepatic impairment.^118,119^ Moreover, rivaroxaban, although effective, exhibits dose-dependent variability in bioavailability. At the 10 mg dose, it achieves 92–98% bioavailability, whereas at 15–20 mg in the fasting state, bioavailability decreases to 66% due to dissolution and absorption limitations.^120–122^

In this context, there were several propositions for transdermal formulations of DOACs in the reviewed studies. Transdermal delivery offers several advantages, including controlled and prolonged drug release leading to reduced dosing frequency, avoidance of gastrointestinal enzymatic degradation and pH-related inactivation, the ability to bypass hepatic first-pass metabolism, and reduced fluctuations in plasma drug concentrations. Transdermal systems can also minimize gastrointestinal toxicity associated with some DOACs and allow therapy to be discontinued immediately in case of adverse reactions.^76,123^

In 2021, Araujo et al. proposed a microemulsion-based hydrogel for transdermal application of rivaroxaban.^76^ Results demonstrated satisfactory drug release, permeation profiles, stability, skin compatibility, and significant anticoagulant activity in vitro. El-Shenawy et al. explored an ethosomal thermoreversible in situ gel for the nasal delivery of apixaban, designed to remain in a liquid state at room temperature and undergo gelation upon contact with the nasal cavity’s body temperature (approximately 32.3 °C). The authors reported that an optimized ethosomal vesicle formulation could provide high drug entrapment efficiency, improved bioavailability, and effective permeation through the nasal mucosa. This apixaban formulation addressed issues related to the low permeability and bioavailability of apixaban by choosing the nasal route to bypass first-pass metabolism.^52^ Vesicular carrier systems, such as liposomes, niosomes, and ethosomes, are suitable for both hydrophilic and lipophilic drugs, serving as reservoirs that control the release rate, protecting the encapsulated drug from environmental factors, and enabling targeted drug delivery to specific sites.^124^ Abdulbaqi et al. developed an ultrafine O/W nanoemulsion with permeation-enhancing properties. This formulation generated nanosized droplets (>50 nm) that functioned as permeation enhancers through the skin barrier, along with other formulation excipients. Results indicate significantly enhanced permeability of ultrafine apixaban nanoemulsions compared to pure apixaban, with complete permeation observed in the nanoemulsion formulation.^77^

These studies discussed innovative formulations of the DOACs rivaroxaban and apixaban, contributing to the advancement of nonoral anticoagulant formulations.^52,76,77^ Focused on drug delivery systems through alternative routes rather than the oral route, all formulations were developed to avoid first-pass metabolism, addressing issues such as low bioavailability, gastrointestinal effects, and oral administration difficulties, such as in elderly patients. Although these studies suggest potential use of an alternative system to administer DOACs, formulation development is still in the initial phase, with remaining gaps in long-term stability, consistent drug delivery, and a deeper understanding of efficacy and safety in vivo and humans.

### Other Agents

Studies on transdermal thrombosis treatment also considered APIs such as hirudin and fucoidan. Hirudin, a polypeptide derived from the salivary glands of leeches, exerts its anticoagulant effect by reversibly binding to thrombin, independently of plasma coagulation factors.^80,125,126^ Fucoidan has a sulfated polysaccharide structure, which enhances interaction with coagulation factors, reducing clot formation. Its anticoagulant effect occurs by inhibiting thrombin formation through both intrinsic and extrinsic pathways of the blood coagulation cascade.^127^

Wu et al. proposed a r-hirudin-loaded and hyaluronic acid (HA)-based microneedle to achieve transdermal drug delivery.^79^ According to this study, the new formulation could significantly prevent thromboembolic disease without bleeding in animal models. Findings show that 3D-printed microneedles with r-hirudin can enable customizable, personalized transdermal anticoagulant delivery for the minimally invasive, long-term treatment of thrombotic disease.

In addition to hirudin’s anticoagulant mechanism, Men et al. developed a recombinant hirudin incorporating an Arg-Gly-Asp (RGD) sequence, which competitively inhibits the binding of fibrinogen to its receptors.^80^ The recombinant hirudin-loaded polymeric microneedle patch demonstrated significant in vivo antithrombotic activity. In rats, peak values of activated partial thromboplastin time (aPTT), thrombin time (TT), and prothrombin time (PT) increased by 1.50-, 1.14-, and 1.17-fold, respectively, compared to the control group. Furthermore, there was no significant difference in peak prolongation times when compared to subcutaneous injections of recombinant hirudin.^80^ Commonly used as clinical laboratory assays, these parameters together provide a comprehensive assessment of anticoagulant activity, as their prolongation indicates reduced efficiency of clot formation and confirms the systemic effects of anticoagulant.^128,129^

Stephanie et al.’s study presents fucoidan-incorporated dissolving microneedles (FC-DMN) as an innovative transdermal anticoagulant delivery system.^78^ The FC-DMN manufactured with gelatin and PVP demonstrated proper stability, mechanical strength, and skin penetration. Compared with conventional heparin injections and gels, FC-DMN achieved high drug permeation (91.23%) and similar anticoagulant effects while ensuring painless, noninvasive administration. The proposed technology could overcome the bioavailability issues of oral and injectable fucoidan by facilitating systemic absorption through the skin.

As observed for heparin studies, microneedles seem particularly explored as a promising alternative for transdermal delivery in fucoidan and hirudin transdermal formulations. Both hirudin and fucoidan are large biomolecules with poor membrane permeability, which makes their systemic delivery via noninvasive methods difficult. Advanced drug delivery technologies, such as microneedles, are under investigation to overcome these challenges and enhance therapeutic efficacy. Challenges such as storage conditions affecting microneedle integrity and long-term drug stability still require investigation.

### Clinical Studies of Transdermal Formulations for Thrombotic Disorders

Additional search of clinical trials on the ClinicalTrials.gov website resulted in a list of 808 studies without applying any date filters. After analyzing the data provided by the website database, none of the 808 studies were related to a known anticoagulant, antiplatelet, or thrombolytic molecule.

This result is consistent with the findings of the presented systematic review, which indicate that significant limitations and challenges remain for the advancement of transdermal drug technologies. One of the major obstacles to transdermal delivery can be attributed to the very specific physicochemical properties required for drugs—such as low molecular weight, proper lipophilicity, low melting point, and high potency.^130,131^ Most of the molecules evaluated in the current studies are pre-existing drugs that were not originally designed for this route, making it unlikely that they have the necessary properties to cross the skin barrier through passive diffusion. Innovations in active drug delivery (e.g., mechanical, thermal or electrical methods, such as microneedles and velocity-based devices, laser and iontophoresis, respectively; or even innovative microelectronic devices) and an approach change in selecting existing drug molecules, as well as design of new molecular entities focusing on transdermal route, would enhance the applicability of the transdermal route as a viable strategy for drug administration.^132^

## Conclusion

Recent advancements in transdermal antithrombotic delivery systems indicate a strong focus on ASA and heparin for alternative skin-based administration, whose interest may be attributed to their continuous therapeutic relevance and their compatibility with controlled-release technologies. Other antithrombotic agents considered in studies were fucoidan, hirudin, rivaroxaban, and apixaban. Research efforts targeted several key challenges in current treatments, such as short half-lives, reduced bioavailability, the need for dosage monitoring, discomfort associated with injections, and bleeding risks. Advanced controlled-release systems, painless transdermal administration, and reduced bleeding risks through “smart” formulations are among the strategies proposed to overcome these obstacles. A variety of microneedles seem to be an effective and promising solution for transdermal delivery of antithrombotic molecules. There is growing potential to expand the types of drugs that can be delivered effectively across the skin. As microneedles, the refinement of active delivery methods could increase the importance and commercial value of transdermal delivery systems. In parallel, a rational drug candidate selection or design with a focus on transdermal administration could be a strategic direction in early stage pharmaceutical development to enable more drugs with necessary characteristics. Future studies will be necessary to support the design and optimization of scalable, effective, and cost-effective products as well as to address potential regulatory concerns over the use of innovative transdermal formulations and devices.

## Methods

This systematic review took place under the Preferred Reporting Items for Systematic Reviews and Meta-Analyses (PRISMA) guidelines.^133^ The literature search was in Web of Science, ScienceDirect, and PubMed databases, utilizing keywords from titles and abstract fields and adding the Boolean connector “AND” to every search to ensure that all retrieved results contained every specified keyword. Search terms were classified into three categories (Table 2). All combinations of terms from groups 1, 2, and 3 were applied: group 1 referred to skin drug delivery routes; group 2 referred to delivery systems and formulations; and group 3 referred to antithrombotic agents and classes. Only original research articles published in English between 2014 and the date of submission (2025) were included. The analysis excluded other publications, such as conference papers, reviews, and case reports.

**Table 2:** Search Term Categories

group 1 drug delivery routes	group 2 delivery systems and formulations	group 3 antithrombotic agents and classes
transdermal	patch	anticoagulant
cutaneous	hydrogel	rivaroxaban
skin	ointment	apixaban
topical	cream	edoxaban
transmucosal	microneedle	dabigatran
	film	aspirin
	gel	clopidogrel
	paste	heparin
	membrane	enoxaparin
	microemulsion	warfarin
	nanoparticle	factor Xa inhibitor
	nanocarrier	direct thrombin inhibitor
		fondaparinux
		tenecteplase
		alteplase
		thrombolytic agent
		fibrinolytic agent

In order to contribute to this review study on the advances in TDDSs for antithrombotic therapy, an additional search was conducted in April of 2025 on the ClinicalTrials.gov website using the keyword “transdermal” in the “treatment/intervention” field, with the studies status filter set to “active”, “not recruiting”, “completed”, and “terminated”. *ClinicalTrials.gov* is a website and online database of clinical research studies, maintained by the US National Library of Medicine. According to website information, its purpose is to provide information about clinical research studies to the public, researchers, and healthcare professionals.^134^

## Abbreviations

ADP: adenosine diphosphate
API: active pharmaceutical ingredients
aPTT: activated partial thromboplastin time
ASA: acetylsalicylic acid or aspirin
ATCCS: anatomical therapeutic chemical classification system
CAD: coronary artery disease
COVID-19: Coronavirus Disease 2019
COX: cyclooxygenase
DOACs: direct oral anticoagulants
FC-DMN: fucoidan-incorporated dissolving microneedles
GP: glycoprotein
GPCRs: G protein–coupled receptors
HA: hyaluronic acid
LMWH: low-molecular-weight heparin
NIR: near-infrared
O/W: oil/water
PRISMA: preferred reporting items for systematic reviews and meta-analyses
PS: phosphatidylserine
PT: prothrombin time
PVA: poly­(vinyl alcohol)
PVP: polyvinylpyrrolidone
TDDS: transdermal drug delivery systems
TF: tissue factor
tPA: tissue plasminogen activator
TT: thrombin time
TxA2: thromboxane A2
UFH: unfractionated heparin
UV: ultraviolet
VTE: venous thromboembolism
WHO: World Health Organization
